# Preoperative Predictors of Subsequent Breast Cancer Events Detected on Abbreviated MRI in Patients with Early-Stage Breast Cancer

**DOI:** 10.3390/diagnostics15232953

**Published:** 2025-11-21

**Authors:** Na Lae Eun, Ji Hyun Youk, Jeong-Ah Kim, Yoon Jin Cha, Soong June Bae, Sung Gwe Ahn, Joon Jeong, Hyejin Yang, Hye Sun Lee, Eun Ju Son

**Affiliations:** 1 Department of Radiology, Gangnam Severance Hospital, Yonsei University College of Medicine, Seoul 06273, Republic of Korea; enrlove@yuhs.ac (N.L.E.);; 2 Department of Pathology, Gangnam Severance Hospital, Yonsei University College of Medicine, Seoul 06273, Republic of Korea; 3 Department of Surgery, Gangnam Severance Hospital, Yonsei University College of Medicine, Seoul 06273, Republic of Korea; 4 Biostatistics Collaboration Unit, Yonsei University College of Medicine, Seoul 03722, Republic of Korea

**Keywords:** preoperative predictors, subsequent breast cancer events, breast cancer recurrence, abbreviated breast MRI protocols, surveillance, early-stage breast cancer

## Abstract

**Background/Objectives**: This study aimed to investigate the preoperative clinicopathologic and imaging features associated with subsequent breast cancer events detected on postoperative abbreviated MRI in early-stage breast cancer patients following breast and axillary surgery. **Methods:** A retrospective analysis was conducted on 1171 patients (median age, 53 years; range, 24–90 years) diagnosed with clinical stage I or II breast cancer between January 2013 and December 2017. Logistic regression analysis was used to evaluate preoperative imaging features—including breast density assessed on mammography and MRI descriptors—along with clinicopathologic characteristics, to identify factors independently associated with subsequent breast cancer events during abbreviated MRI screening. **Results:** Among the patients, 57 (4.9%) experienced subsequent breast cancer events at a median follow-up of 74 months. In the multivariable analysis, high nuclear grade (odds ratio [OR] = 2.821; 95% confidence interval [CI], 1.427–5.577; *p* = 0.003), dense breast tissue on mammography (OR = 4.680; 95% CI, 1.113–19.684; *p* = 0.035), and absence of heterogeneous internal enhancement on preoperative MRI (OR = 0.429; 95% CI, 0.206–0.891; *p* = 0.023) were independently associated with subsequent breast cancer events detected using an abbreviated breast MRI protocol. Age ≥ 40 years (OR = 0.448; 95% CI, 0.193–1.039; *p* = 0.061) and clinical T2 stage (OR = 1.744; 95% CI, 0.969–3.139; *p* = 0.064) showed borderline significance. **Conclusions:** High nuclear grade, dense breast tissue on mammography, and absence of heterogeneous internal enhancement on preoperative MRI were associated with an increased risk of subsequent breast cancer events in patients undergoing abbreviated MRI surveillance following surgery for early-stage breast cancer.

## 1. Introduction

Breast cancer patients with a personal history of breast cancer (PHBC) carry a higher risk of developing secondary breast cancers, which is an independent predictor of breast cancer survival related to distant metastasis or breast cancer-related mortality [1,2,3]. Annual mammography has long been the standard post-treatment imaging modality for breast cancer surveillance, contributing to significant improvements in patient outcomes [4,5]. However, despite its effectiveness, annual mammography surveillance for breast cancer survivors has been reported to be less effective than in those without a prior history [6]. Moreover, breast density further limits surveillance efficacy, as extremely dense breasts yield lower sensitivity and increased interval cancers [7], highlighting the challenges in early detection for patients with PHBC.

Breast MRI is widely recognized for its high sensitivity and superior cancer detection rates compared with mammography and ultrasound (US). Prior studies have reported MRI sensitivity exceeding 90%, significantly outperforming other modalities in both screening and diagnostic settings [8,9,10]. While the use of breast MRI for patients without a high familial risk remains a topic of debate, several studies have advocated for its use as a screening tool in patients with PHBC or in women with dense breast tissue [11,12,13,14,15,16,17]. Additionally, the American College of Radiology guidelines recommend annual MRI surveillance for patients diagnosed with dense breast tissue or those under 50 years of age who have a personal history of breast cancer [18]. In this context, abbreviated breast MRI has emerged as a streamlined alternative to full diagnostic protocols, offering reduced acquisition and interpretation time while maintaining high sensitivity and demonstrating noninferiority in both screening and postoperative surveillance settings [19,20,21,22,23].

Nevertheless, data on subsequent breast cancer events after postoperative screening MRI remain scarce. Several studies have explored outcomes and factors associated with recurrence or interval cancers, particularly those with a PHBC [13,16,17]. These studies identified risk factors such as being under the age of 50 and previous treatment for ductal carcinoma in situ as contributors to second breast cancers. While these findings are informative, they do not address whether preoperative MRI features might influence postoperative surveillance outcomes, particularly in the setting of abbreviated MRI protocols. Determining the patient subgroups appropriate for postoperative abbreviated MRI surveillance is an important clinical and research question, with implications for developing risk-adapted and individualized surveillance strategies.

Therefore, this study aimed to identify preoperative clinicopathologic and imaging factors associated with subsequent breast cancer events that were detected on postoperative abbreviated MRI surveillance in patients with early-stage breast cancer.

## 2. Materials and Methods

### 2.1. Study Population

**Figure 1:**
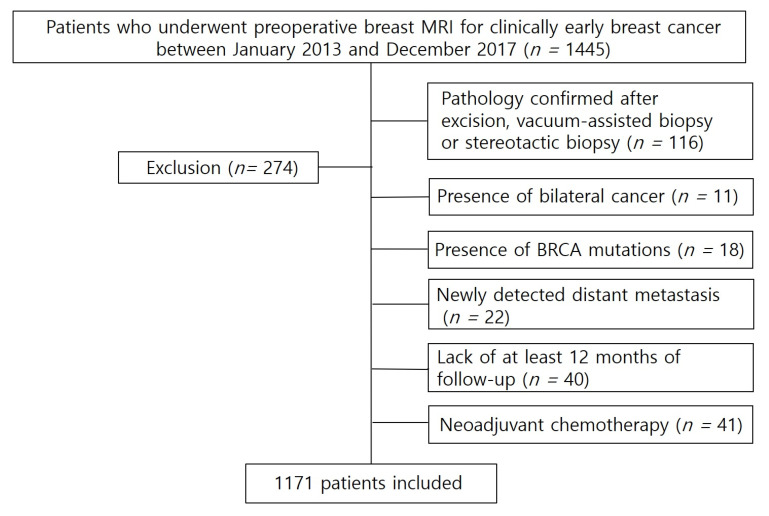
Flowchart of the study population.

This retrospective study was approved by the Institutional Review Board of Gangnam Severance Hospital (Approval Code: 2023-0149-001; Approval Date: 14 April 2023), and the requirement to obtain patient consent or written informed consent was waived. Since 2013, breast MRI has been incorporated into the routine post-treatment surveillance protocol at our institution, a tertiary referral university hospital for patients who have undergone definitive breast cancer surgery (breast-conserving surgery or mastectomy). According to our institutional postoperative surveillance protocol, the first breast MRI was performed approximately 12 months after curative breast and axillary surgery, followed by annual imaging thereafter. Our practice also involves annual mammography and breast US every 6 to 12 months. Between January 2013 and December 2017, a total of 1445 female patients with clinically early-stage invasive breast cancer (clinical T1–T2 and N0) underwent preoperative evaluation with US and MRI, followed by surgery. Of these, 274 were excluded due to diagnosis by excisional or vacuum-assisted biopsy rather than core needle biopsy, prior or bilateral breast cancer, BRCA mutation, neoadjuvant chemotherapy, or insufficient follow-up duration. Finally, a total of 1171 patients were included (Figure 1).

### 2.2. MRI Technique

MRI examinations were conducted using 3-T systems (Achieva; Philips Medical Systems and Discovery MR750; General Electric Medical Systems), with a dedicated breast coil. All imaging was performed with bilateral axial views. The standard imaging protocol included turbo spin-echo T1-weighted (repetition time/echo time [in milliseconds], 505/10; matrix, 564 × 338; field of view, 20–34 cm; slice thickness, 3 mm) and T2-weighted sequences with fat suppression (repetition time/echo time [ms], 5506/70; matrix, 564 × 261; field of view, 20–34 cm; slice thickness, 3 mm). Dynamic contrast-enhanced MRI comprised one pre-contrast and five post-contrast series, using a fat-suppressed T1-weighted gradient-echo sequence (repetition time/echo time [ms], 5/2.5; matrix, 340 × 274; flip angle, 12°; field of view, 34 cm; sliced thickness, 2 mm). The contrast agents used were either Gadopentetate Dimeglumine (Bono-I; Central Medical Service, Seoul, South Korea) or Gadobutrol (Gadovist; Bayer Healthcare, Berlin, Germany) at a dose of 0.1 mmol/kg body weight, administered with an automated injector (Nemoto; Nemoto Kyorindo, Tokyo, Japan) at a rate of 2 mL/s, followed by a 20 mL saline flush. For the abbreviated MRI protocol analysis, only the relevant sequences were retrospectively selected from the full diagnostic protocol. The abbreviated protocol consisted of (1) a T2-weighted fat-suppressed spin-echo sequence, (2) one pre-contrast T1-weighted sequence, and (3) the first post-contrast T1-weighted dynamic sequence, including subtraction images and maximum-intensity-projection (MIP) reconstruction. This selection was based on previously published abbreviated MRI protocols demonstrating noninferior diagnostic performance compared with full diagnostic protocols [24].

### 2.3. Image Analysis

All preoperative mammography and MR studies were interpreted by two experienced radiologists with 25 and 9 years of dedicated breast imaging experience, who reached a consensus based on the fifth edition of the Breast Imaging Reporting and Data System (BI-RADS) classification system, without access to the patients’ clinical histories or histopathologic results to minimize bias. For the purpose of abbreviated MRI analysis, we retrospectively selected and reviewed sequences from full diagnostic MRI scans that corresponded to a typical abbreviated MRI protocol. Specifically, the analysis was limited to the T2-weighted sequence, one pre-contrast T1-weighted sequence, and the first post-contrast T1-weighted dynamic sequence, including subtraction images and MIP reconstruction.

On mammography, breast density was assessed according to the ACR BI-RADS categories and dichotomized into non-dense (A or B) and dense (C or D) breasts for analysis. Dense breast tissue was defined as BI-RADS category C or D, indicating heterogeneously or extremely dense breasts that could obscure small masses.

On preoperative MRI, the amount of fibroglandular tissue (FGT; categorized as non-dense or dense) and the level of background parenchymal enhancement (BPE; classified as minimal or mild, moderate, or marked) were assessed. The morphologic characteristics of each tumor lesion, including the presence of associated non-mass enhancement (NME), were evaluated. For masses, shape (oval, round, or irregular), margins (circumscribed, irregular, or spiculated), and internal enhancement characteristics (homogeneous; confluent uniform enhancement, heterogeneous; nonuniform enhancement with variable signal intensity, rim enhancement; more pronounced enhancement at the periphery of the mass, or dark internal septations; dark, nonenhancing lines within a mass) were evaluated. Heterogeneous enhancement was defined as a non-uniform internal enhancement with mixed or irregular signal intensity, reflecting variable vascularity and contrast uptake within the lesion. For NMEs, the distribution (focal, linear, segmental, regional, multiple-regional, diffuse) and internal enhancement characteristics (homogeneous, heterogeneous, clumped, or clustered-ring enhancement) were analyzed. Additionally, intratumoral T2 high signal intensity and peritumoral edema were assessed. Intratumoral T2-high-signal intensity was identified when the tumor signal intensity was higher than the surrounding tissue or comparable to water or vessels. Peritumoral edema was defined as high signal intensity on T2-weighted images, either behind the tumor in the pre-pectoral area or around the tumor mass. The clinical T stage was assessed as T1 (tumor < 2 cm) and T2 (tumor ≥ 2 cm and <5 cm).

### 2.4. Histopathologic Assessment

The pathologic assessment was performed on core-needle biopsy specimens obtained before surgery by a pathologist with 10 years of experience in breast pathology. The pathologic tumor size, histologic type, nuclear grade, presence of lymphovascular invasion (LVI), and molecular subtype, including the expression of estrogen receptor (ER), progesterone receptor (PR), and HER2, were evaluated using the standard avidin–biotin complex immunohistochemical staining method. ER and PR positivity were determined using the Allred score, which rates the proportion of positive cells and staining intensity; cases with an Allred score greater than 3 were considered positive. Tumors were defined as HER2-positive when the HER2 status was 3+. When HER2 was equivocal (2+) by immunohistochemistry (IHC), gene amplification using fluorescence in situ hybridization analysis or silver in situ hybridization analysis was performed. Tumors were classified into three subtypes: hormone-positive type (ER- or PR-positive, HER2-negative or positive), HER2-positive (ER- and PR-negative, HER2-positive), and triple-negative type (ER- and PR-negative, HER2-negative).

### 2.5. Statistical Analysis

The primary endpoint of this study was subsequent breast cancer events detectable on postoperative abbreviated MRI protocol, defined as either second breast cancer (ipsilateral breast cancer following breast-conserving surgery or contralateral breast cancer) or locoregional recurrence involving the ipsilateral mastectomy bed, axillary, supraclavicular, infraclavicular, or internal mammary lymph nodes. All subsequent breast cancer events were pathologically confirmed whenever tissue sampling was performed. In cases without available histopathologic confirmation, diagnosis was based on concordant multimodality imaging findings and documented clinical progression during follow-up. The clinicopathologic characteristics of the patients and their initial breast cancers were compared based on the status of subsequent breast cancer events. Categorical variables were compared using the chi-square test or Fisher’s exact test, whereas continuous variables were analyzed using the Mann–Whitney U test. Logistic regression analyses were performed to identify preoperative factors associated with subsequent breast cancer events. Clinically relevant variables showing potential associations in the univariable analysis (*p* < 0.10), together with established clinicopathologic features, were included in the multivariable logistic regression model using the enter method. Odds ratios (ORs) with 95% confidence intervals (CIs) were calculated, and *p*-values < 0.05 were considered statistically significant. The OR represented the relative odds of developing subsequent breast cancer associated with a given variable compared with a reference group. An OR greater than 1 indicates an increased risk, whereas an OR less than 1 indicates a decreased risk. The reference categories (Ref) were chosen as the lower-risk or baseline groups to allow intuitive interpretation of odds ratios. A two-sided *p*-value < 0.05 was considered statistically significant. All statistical analyses were conducted using SAS version 9.4 (SAS Institute; Cary, NC, USA).

## 3. Results

A total of 1171 female patients diagnosed with early-stage breast cancer (stage I or II) were included in this study. The median age was 53 years (range, 24–90 years). During a median follow-up period of 74 months (interquartile range, 60–97 months), 57 patients (4.9%) experienced subsequent breast cancer events, including second breast cancers or locoregional recurrences detected during postoperative abbreviated breast MRI surveillance. The events comprised 28 s breast cancers (15 ipsilateral and 13 contralateral) and 29 locoregional events involving the ipsilateral chest wall (involving the mastectomy bed or adjacent soft tissues; *n* = 15), ipsilateral axilla (*n* = 12), internal mammary (*n* = 1), and supraclavicular lymph nodes (*n* = 1).

Among the clinicopathologic characteristics (Table 1), patients who experienced subsequent breast cancer events were more likely to be younger than 40 years (14.4% vs. 6.0%, *p* = 0.025), to have a high nuclear grade (57.9% vs. 34.4%, *p* = 0.001), and to show lymphovascular invasion (LVI) (35.1% vs. 20.5%, *p* = 0.03). Regarding imaging features (Supplementary Table S1A), breast density assessed on mammography was significantly associated with an increased subsequent breast cancer risk (dense breast tissue, 96.5% vs. 85.3%, *p* = 0.018) and the proportion of regional distribution on MRI was also significantly higher in patients who experienced subsequent breast cancer events compared with those who did not (47.1% vs. 13.3%, *p* = 0.01). Among treatment variables (Supplementary Table S1B), adjuvant chemotherapy was more frequently administered to patients who developed subsequent breast cancer events (63.2% vs. 45.7%, *p* = 0.01). Other factors, including clinical T stage, lymph node status, hormone receptor status, and molecular subtype classification, were not significantly associated (all *p* > 0.05).

**Figure 2:**
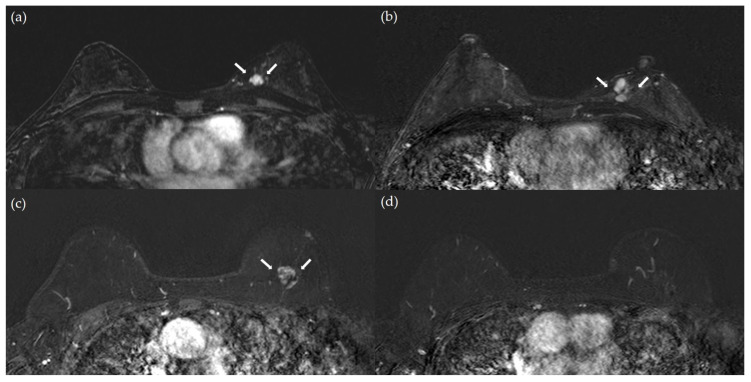
Representative cases of a subsequent breast cancer event and a non-subsequent breast cancer event: (**a**,**b**) A 28-year-old woman with ER/PR-positive, HER2-negative breast cancer and high nuclear grade. (**a**) Preoperative contrast-enhanced T1-weighted MR image with subtraction shows a 1.5 cm irregular, homogeneously enhancing mass in the upper medial quadrant of the left breast (arrows). (**b**) Postoperative contrast-enhanced T1-weighted MR image with subtraction obtained 60 months later reveals a new irregular mass in the medial central portion of the left breast (arrows), confirmed as a subsequent breast cancer event by core needle biopsy. (**c**,**d**) A 58-year-old woman with ER/PR-positive, HER2-negative breast cancer without high nuclear grade. (**c**) Preoperative T1-weighted MR image with subtraction shows a 2.0 cm irregular mass in the upper medial quadrant of the left breast with heterogeneous internal enhancement, characterized by nonuniform signal intensity and intermixed enhancing and nonenhancing areas (arrows). (**d**) Postoperative contrast-enhanced T1-weighted MR image obtained 60 months later shows no evidence of subsequent breast cancer event.

To identify independent preoperative predictors of subsequent breast cancer risk, univariable and multivariable logistic regression analyses were performed using only preoperatively available clinicopathologic and imaging factors (Table 2 and Table 3). In univariable analysis, several variables were significantly associated with the risk of increased subsequent breast cancer, including younger age (<40 years) (OR = 0.392; 95% CI, 0.178–0.861; *p* = 0.0197) and high nuclear grade (OR = 2.617; 95% CI, 1.525–4.492; *p* = 0.0005) (Figure 2). Dense breast tissue assessed on mammography (BI-RADS C/D) was also associated with an increased risk of subsequent breast cancer (OR = 4.747; 95% CI, 1.147–19.649; *p* = 0.0316). Conversely, heterogeneous internal enhancement on preoperative MRI was inversely associated with subsequent breast cancer risk (OR = 0.451; 95% CI, 0.226–0.901; *p* = 0.0242). Patients with clinical T2 stage tended to have a higher risk of subsequent breast cancer events than those with T1 stage, although the association did not reach statistical significance (OR = 1.652; 95% CI, 0.968–2.819; *p* = 0.0658). Similarly, patients with HER2-positive tumors showed a trend toward an increased risk compared with those with HER2-negative tumors (OR = 1.697; 95% CI, 0.951–3.029; *p* = 0.0734). ER, PR, molecular subtype, and other MRI features were not significant predictors.

In multivariable analysis (Table 3), high nuclear grade (OR = 2.821; 95% CI, 1.427–5.577; *p* = 0.003), indicating that patients with high-grade tumors had approximately 2.8 times higher odds of developing subsequent breast cancer compared with those with low- or intermediate-grade tumors and the presence of dense breast tissue assessed on mammography (OR = 4.680; 95% CI, 1.113–19.684; *p* = 0.035) remained independent preoperative predictors of increased subsequent breast cancer risk. Heterogeneous internal enhancement on preoperative MRI continued to show a protective effect (OR = 0.429; 95% CI, 0.206–0.891; *p* = 0.023). Age ≥ 40 years (OR = 0.448; 95% CI, 0.193–1.039; *p* = 0.061) and clinical T2 stage (OR = 1.744; 95% CI, 0.969–3.139; *p* = 0.064) showed borderline significance.

Notably, in the univariable analysis, HER2 status was initially associated with increased subsequent breast cancer risk. However, this association did not remain significant in the multivariable model, suggesting that its effect may be partly explained or confounded by other variables. In contrast, the presence of high nuclear grade, dense breast tissue assessed on mammography, and heterogeneous internal enhancement pattern on MRI maintained statistical significance even after adjusting for multiple preoperative variables, confirming their roles as independent preoperative predictors of subsequent breast cancer risk.

## 4. Discussion

The findings of our study identify factors associated with subsequent breast cancer events in early-stage breast cancer patients who underwent postoperative abbreviated MRI surveillance. Our results highlight several independent predictors of subsequent breast cancer events, including high nuclear grade, dense breast tissue on mammography, and absence of heterogeneous internal enhancement of the tumor on preoperative MRI.

Dense breast tissue on mammography was an independent predictor of subsequent breast cancer events (OR = 4.680; 95% CI, 1.113–19.684; *p* = 0.035), supporting the role of breast density assessed on mammography as a significant preoperative risk factor. This observation is consistent with previous guidelines recommending postoperative MRI for patients with a PHBC who have dense breast tissue [18,25]. While our findings did not reach statistical significance, they support the consideration of breast density in risk assessment models to help identify patients who may benefit from postoperative abbreviated MRI surveillance. Furthermore, because breast density varies across ethnic groups—with Asian women generally exhibiting higher density than Western populations [26,27]—the prognostic impact of breast density may differ by population. This possibility warrants validation in larger, multi-ethnic prospective studies to confirm generalizability and to clarify the role of breast density in tailoring surveillance strategies.

Notably, the presence of ‘heterogeneous internal enhancement’ (nonuniform enhancement with variable signal intensity) on preoperative MRI was associated with a reduced risk of subsequent breast cancer events (OR = 0.429; 95% CI, 0.206–0.891; *p* = 0.023). This finding suggests that the absence of heterogeneous internal enhancement may reflect less aggressive tumor biology and highlights the potential value of preoperative MRI features as additional prognostic indicators beyond conventional clinicopathologic factors. Interestingly, our study contrasts with previous literature suggesting that heterogeneous internal enhancement often reflects biologically aggressive phenotypes and increased subsequent breast cancer risk [28,29,30]. This discrepancy may be explained by the clinical context: all patients in our cohort underwent preoperative MRI for early-stage disease. In this setting, lesions with heterogeneous internal enhancement may have appeared more conspicuous due to internal complexity or vascularity, potentially facilitating earlier detection, more accurate surgical planning, or prompt treatment decisions. Conversely, tumors with homogeneous enhancement may lead to underestimation of disease extent. These differences from prior studies may also reflect methodological and outcome variations. Previous investigations often included patients with more advanced disease, employed full diagnostic MRI protocols, or defined outcomes more broadly to encompass distant metastases and overall survival. By contrast, our study focused exclusively on early-stage breast cancer and evaluated outcomes in the context of postoperative abbreviated MRI surveillance, where heterogeneous internal enhancement may improve lesion conspicuity and facilitate treatment planning. Further prospective studies are warranted to elucidate the prognostic significance of enhancement patterns observed on preoperative MRI in early-stage breast cancer.

The association between high nuclear grade and increased risk of subsequent breast cancer events (OR = 2.821; 95% CI, 1.427–5.577; *p* = 0.003) emphasizes the role of tumor biology in disease progression. High nuclear grade is a marker of more aggressive tumor behavior, which may contribute to the higher likelihood of subsequent breast cancer events observed in this study. Previous studies have also reported that high nuclear grade is significantly associated with an increased risk of recurrence in breast cancer, indicating its prognostic relevance even in early-stage or node-negative disease [31,32]. This finding suggests that patients with high nuclear grade may benefit from abbreviated MRI surveillance postoperatively, offering a personalized approach to postoperative surveillance. Given its reduced scan time, lower cost, and diagnostic efficiency, abbreviated MRI represents a feasible alternative to full protocol MRI in selected clinical scenarios [24].

Numerous prior studies have demonstrated that younger age and larger tumor size are associated with higher risks of breast cancer recurrence. Younger patients tend to exhibit more biologically aggressive tumors and poorer disease-free survival in multiple cohorts [33,34,35]. Additionally, large tumor size has long been established as an adverse prognostic factor in recurrence and survival analyses [36,37]. In our study, age ≥ 40 years (OR = 0.448; 95% CI, 0.193–1.039; *p* = 0.061) and clinical T2 stage (OR = 1.744; 95% CI, 0.969–3.139; *p* = 0.064) showed only borderline associations with subsequent breast cancer events, and tumor size (as captured by clinical T stage) was not independently significant in our multivariable model. This discrepancy may be due to the restricted cohort of early-stage breast cancer patients, which limits the variation in tumor size and disease burden, thereby attenuating the prognostic influence of these factors. Nevertheless, the observed borderline trends are consistent with the established role of age and tumor size in recurrence risk and suggest that they might still carry prognostic value worth further investigation in early-stage breast cancer.

Although molecular subtypes such as HER2-positive and triple-negative breast cancers are well-established predictors of subsequent breast cancer event patterns and clinical outcomes [38,39], their impact was not statistically significant in our analysis. This result is most likely explained by the relatively small number of subsequent breast cancer events within each subtype, particularly in the triple-negative group, which limited statistical power. Validation in larger, subtype-stratified cohorts with longer follow-up will be required to clarify the prognostic role of tumor biology and treatment factors in the context of postoperative abbreviated MRI surveillance.

This study has several limitations. First, its retrospective, single-center design may have introduced selection and interpretation bias and limited generalizability to broader patient populations. Second, although the overall cohort was relatively large, the number of subsequent breast cancer events was small, which reduced statistical power and may have increased the risk of overfitting in the logistic regression model. Larger, prospective studies with sufficient sample sizes are required to validate these associations. Third, abbreviated MRI images were retrospectively reconstructed from full diagnostic protocols, which may affect reproducibility, although the selected sequences align with widely accepted abbreviated protocols. Fourth, patients who received neoadjuvant chemotherapy were excluded to avoid treatment-related alterations in imaging features, and the relatively high proportion of TNBC and HER2-positive cases reflects the practice pattern during the study period, when neoadjuvant therapy was not routinely administered for early-stage disease. Finally, we did not perform survival analyses, which limits conclusions regarding long-term outcomes.

## 5. Conclusions

In conclusion, high nuclear grade and specific preoperative imaging features—including dense breast tissue on mammography and the absence of heterogeneous internal enhancement on MRI—were identified as independent predictors of subsequent breast cancer events in patients with early-stage breast cancer undergoing postoperative abbreviated MRI surveillance. Borderline associations were also observed for older age (≥40 years) and clinical T2 stage. Identifying such preoperative predictors may facilitate individualized risk stratification among early-stage breast cancer survivors, enabling more tailored postoperative surveillance strategies using abbreviated MRI and potentially allowing earlier detection of subsequent breast cancer events.

## Figures and Tables

**Table 1:** Baseline clinicopathologic characteristics according to subsequent breast cancer event status.

	Total (*n* = 1171)	Patients Without Subsequent Breast Cancer Events (*n* = 1114)	Patients With Subsequent Breast Cancer Events (*n* = 57)	*p*-Value
Age				0.025
<40	75 (6.40)	67 (6.01)	8 (14.04)	
≥40	1096 (93.60)	1047 (93.99)	49 (85.96)	
Clinical T stage				0.063
T1	673 (57.47)	647 (58.08)	26 (45.61)	
T2	498 (42.53)	467 (41.92)	31 (54.39)	
Pathology				0.775
Invasive ductal carcinoma	1013 (86.51)	962 (86.35)	51 (89.47)	
Invasive lobular carcinoma	61 (5.21)	59 (5.30)	2 (3.51)	
Others	97 (8.28)	93 (8.35)	4 (7.02)	
Histologic grade				0.094
Low–intermediate	780 (66.61)	744 (66.79)	36 (63.16)	
High	235 (20.07)	218 (19.57)	17 (29.82)	
Unknown	156 (13.32)	152 (16.64)	4 (7.02)	
Nuclear grade				0.001
Low–intermediate	753 (64.30)	729 (65.44)	24 (42.11)	
High	416 (35.53)	383 (34.38)	33 (57.89)	
Unknown	2 (0.17)	2 (0.18)	0 (0.00)	
Lymphovascular invasion				0.03
No	881 (75.23)	846 (75.94)	35 (61.40)	
Yes	248 (21.18)	228 (20.47)	20 (35.09)	
Unknown	42 (3.59)	40 (3.59)	2 (3.51)	
Lymph node metastasis				0.759
No	884 (75.49)	840 (75.40)	44 (77.19)	
Yes	287 (24.51)	274 (24.60)	13 (22.81)	
ER				0.558
Negative	251 (21.45)	237 (21.27)	14 (24.56)	
Positive	919 (78.55)	877 (78.73)	43 (75.44)	
PR				0.122
Negative	403 (34.42)	378 (33.93)	25 (43.86)	
Positive	768 (65.58)	736 (66.07)	32 (56.14)	
p53				0.009
Negative	589 (50.30)	570 (51.17)	19 (33.33)	
Positive	582 (49.70)	544 (48.83)	38 (66.67)	
Ki-67 (%)				0.043
<14	665 (56.79)	640 (57.50)	25 (43.86)	
≥14	506 (43.21)	474 (42.50)	32 (56.14)	
HER2				0.154
Negative	880 (75.15)	842 (75.58)	38 (66.67)	
Positive	253 (21.60)	235 (21.10)	18 (31.58)	
Unknown	38 (3.25)	37 (3.32)	1 (1.75)	
Subtype				0.461
Hormone-positive	921 (78.65)	878 (78.82)	43 (75.44)	
HER2-positive	118 (10.08)	109 (9.78)	9 (15.79)	
Triple negative	125 (10.67)	120 (10.77)	5 (8.77)	
Unknown	7 (0.60)	7 (0.63)	0 (0.00)	

Values are expressed as the mean ± standard deviation or numbers (%). ER, estrogen receptor; HER2, human epidermal growth factor receptor 2; PR, progesterone receptor.

**Table 2:** Univariable logistic regression analysis of preoperative clinicopathologic and imaging features associated with subsequent breast cancer events.

	Univariable	
	Odds Ratio (95% CI)	*p*-Value
Age		
<40	Ref	
≥40	0.392 (0.178–0.861)	0.020
Clinical T stage		
T1	Ref	
T2	1.652 (0.968–2.819)	0.066
Pathology		
Invasive ductal carcinoma	Ref	
Invasive lobular carcinoma	0.582 (0.138–2.460)	0.462
Others	0.824 (0.291–2.335)	0.716
Histologic grade		
Low–intermediate	Ref	
High	1.612 (0.888–2.925)	0.117
Nuclear grade		
Low–intermediate	Ref	
High	2.617 (1.525–4.492)	0.001
ER		
Negative	Ref	
Positive	0.830 (0.447–1.543)	0.556
PR		
Negative	Ref	
Positive	0.657 (0.384–1.125)	0.126
HER2		
Negative	Ref	
Positive	1.697 (0.951–3.029)	0.073
Subtype		
Hormone-positive	Ref	
HER2-positive	1.730 (0.814–3.675)	0.154
Triple-negative	0.873 (0.337–2.261)	0.779
Breast density assessed on mammography		
BI-RADS A or B	Ref	
BI-RADS C or D	4.747 (1.147–19.649)	0.032
Background parenchymal enhancement		
Minimal-mild	Ref	
Moderate-marked	1.264 (0.712–2.244)	0.424
Shape		
Oval	Ref	
Round	0.622 (0.217–1.786)	0.378
Irregular	0.893 (0.464–1.721)	0.736
Margin		
Circumscribed	Ref	
Irregular	1.604 (0.563–4.571)	0.377
Spiculated	0.600 (0.166–2.175)	0.437
Internal enhancement of mass		
Homogeneous	Ref	
Heterogeneous	0.451 (0.226–0.901)	0.024
Rim enhancement	0.493 (0.195–1.247)	0.135
Dark internal septation	0.559 (0.068–4.575)	0.587
T2 hyperintensity		
No	Ref	
Yes	0.693 (0.245–1.962)	0.49
Peritumoral edema		
No	Ref	
Yes	1.095 (0.505–2.373)	0.819
Distribution of nonmass enhancement		
Focal	Ref	
Linear	0.910 (0.196–4.216)	0.904
Segmental	0.308 (0.068–1.404)	0.128
Regional	2.853 (0.770–10.568)	0.117
Multiple regional	1.919 (0.061–60.053)	0.711
Diffuse	1.017 (0.041–25.302)	0.992
Internal enhancement of nonmass enhancement		
Homogeneous	Ref	
Heterogeneous	1.352 (0.164–11.137)	0.779
Clumped	2.805 (0.326–24.172)	0.348
Clustered ring	1.220 (0.107–13.945)	0.873

Ref, reference category; OR, odds ratio; CI, confidence interval; ER, estrogen receptor; PR, progesterone receptor; HER2, human epidermal growth factor receptor 2.

**Table 3:** Multivariable logistic regression analysis of preoperative clinicopathologic and imaging features associated with subsequent breast cancer events.

	Multivariable	
	Odds Ratio (95% CI)	*p*-Value
Age		
<40	Ref	
≥40	0.448 (0.193–1.039)	0.061
Clinical T stage		
T1	Ref	
T2	1.744 (0.969–3.139)	0.064
Histologic grade		
Low–intermediate	Ref	
High	0.684 (0.323–1.448)	0.321
Nuclear grade		
Low–intermediate	Ref	
High	2.821 (1.427–5.577)	0.003
ER		
Negative	Ref	
Positive	1.556 (0.654–3.702)	0.317
PR		
Negative	Ref	
Positive	0.786 (0.364–1.699)	0.541
HER2		
Negative	Ref	
Positive	0.73 (0.089–5.997)	0.770
Breast density assessed on mammography		
BI-RADS A or B	Ref	
BI-RADS C or D	4.68 (1.113–19.684)	0.035
Internal enhancement of mass		
Homogeneous	Ref	
Heterogeneous	0.429 (0.206–0.891)	0.023
Rim enhancement	0.488 (0.184–1.295)	0.150
Dark internal septations	0.504 (0.058–4.34)	0.533

Ref, reference category; OR, odds ratio; CI, confidence interval.

## Data Availability

The data presented in this study are available upon request from the corresponding author. The data are not publicly available due to confidentiality restrictions.

## Supplementary Materials

The following supporting information can be downloaded at: https://www.mdpi.com/article/10.3390/diagnostics15232953/s1, Supplementary Table S1A. Baseline imaging characteristics according to subsequent breast cancer event status. Supplementary Table S1B. Baseline treatment information according to subsequent breast cancer event status.

## Abbreviations

The following abbreviations are used in this manuscript:

BPE	Background Parenchymal Enhancement
CI	Confidence Interval
ER	Estrogen receptor
HER2	Human epidermal growth factor receptor 2
IHC	I Immunohistochemistry
LVI	Lymphovascular invasion
MRI	Magnetic resonance imaging
PHBC	Personal history of breast cancer
PR	Progesterone receptor
