# Supplementary material for: Preoperative Predictors of Subsequent Breast Cancer Events Detected on Abbreviated MRI in Patients with Early-Stage Breast Cancer
Source: Diagnostics (Basel). 2025 Nov 21;15(23):2953. doi: 10.3390/diagnostics15232953 (PMC12691065; doi:10.3390/diagnostics15232953)
Supplement: Supplementary file 1 [file diagnostics-15-02953-s001.zip › diagnostics-3918002-supplementary.pdf]

Supplementary material

Supplementary Table S1-A. Baseline imaging characteristics according to subsequent breast cancer event status

	Total (n=1171)	Patients without subsequent breast cancer events (n=1114)	Patients with subsequent breast cancer events (n=57)	p-value
Breast density assessed on mammography				0.018
BI-RADS A or B	166 (14.18)	164 (14.72)	2 (3.51)	
BI-RADS C or D	1005 (85.82)	950 (85.28)	55 (96.49)	
Background parenchymal enhancement				0.423
Minimal-mild	855 (73.01)	816 (73.25)	39 (68.42)	
Moderate-marked	316 (26.99)	298 (26.75)	18 (31.58)	
Shape (n=1012)				0.673
Oval	220 (21.74)	207 (21.58)	13 (24.53)	
Round	133 (13.14)	128 (13.35)	5 (9.43)	
Irregular	659 (65.12)	624 (65.07)	35 (66.04)	
Margin (n=1012)				0.057
Circumscribed	98 (9.68)	94 (9.80)	4(7.55)	
Irregular	673 (66.50)	630 (65.69)	43 (81.13)	
Spiculated	241 (23.81)	235 (24.50)	6 (11.32)	
Internal enhancement of mass (n=1012)				0.145
Homogeneous	126 (12.45)	114 (11.89)	12 (22.64)	
Heterogeneous	706 (69.76)	674 (70.28)	32 (60.38)	
Rim-enhancement	162 (16.01)	154 (16.06)	8 (15.09)	
Dark internal septation	18 (1.78)	17 (1.77)	1 (1.89)	
T2 hyperintensity (n=1012)				0.488
No	907 (89.62)	858 (89.47)	49 (92.45)	
Yes	105 (10.38)	101 (10.53)	4 (7.55)	
Peritumoral edema (n=1012)				0.819
No	870 (85.97)	825 (86.03)	45 (84.91)	
Yes	142 (14.03)	134 (13.97)	8 (15.09)	
Associated nonmass enhancement (n=1012)				0.928
No	769 (75.99)	729 (76.02)	40 (75.47)	
Yes	243 (24.01)	230 (23.98)	13 (24.53)	
Distribution of nonmass enhancement (n=402)				0.010
Focal	63 (15.67)	60 (15.58)	3 (17.65)	
Linear	69 (17.16)	66 (17.14)	3 (17.65)	
Segmental	199 (49.50)	196 (50.91)	3 (17.65)	
Regional	59 (14.68)	51 (13.25)	8 (47.06)	
Multiple regional	4 (1.00)	4 (1.04)	0 (0.00)	
Diffuse	8 (1.99)	8 (2.08)	0 (0.00)	

	Total (n=1171)	Patients without subsequent breast cancer events (n=1114)	Patients with subsequent breast cancer events (n=57)	p-value
Internal enhancement of nonmass enhancement (n=402)				0.544
Homogeneous	37 (9.20)	36 (9.35)	1 (5.88)	
Heterogeneous	221 (54.98)	213 (55.32)	8 (47.06)	
Clumped	83 (20.65)	77 (20.00)	6 (35.29)	
Clustered ring	61 (15.17)	59 (15.32)	2 (11.76)	

Values are expressed as the mean ± standard deviation or number (%).

Supplementary Table S1-B. Baseline treatment information according to subsequent breast cancer event status

	Total (n=1171)	Patients without subsequent breast cancer events (n=1114)	Patients with subsequent breast cancer events (n=57)	p-value
Surgery type				0.206
Total mastectomy	481 (41.08)	453 (40.66)	28 (49.12)	
Breast conserving surgery	690 (58.92)	661 (59.34)	29 (50.88)	
Adjuvant chemotherapy				0.010
No	626 (53.46)	605 (54.31)	21 (36.84)	
Yes	545 (46.54)	509 (45.69)	36 (63.16)	
Adjuvant endocrine therapy				0.98
No	204 (17.42)	194 (17.41)	10 (17.54)	
Yes	967 (82.58)	920 (82.59)	47 (82.46)	
Adjuvant radiation therapy				0.322
No		454 (40.75)	27 (67.37)	
Yes		660 (59.25)	30 (52.63)	

Values are expressed as the mean ± standard deviation or number (%).
